# Relationship Between Quantitative MRI UTE T2* of ACL Autografts and BMI-Normalized Knee Laxity Within the First Year After ACL Reconstruction

**DOI:** 10.1177/03635465251368393

**Published:** 2025-09-19

**Authors:** Alonso Figueroa, Tomasz Bugajski, Dillon Humpal, Manickam Kumaravel, Walter Lowe, Payam Zandiyeh

**Affiliations:** * Department of Orthopedic Surgery, University of Texas, Health Science Center at Houston, Houston, Texas, USA; † Department of Diagnostic and Interventional Radiology, University of Texas, Health Science Center at Houston, Houston, Texas, USA; Investigation performed at the University of Texas Health Science Center, Houston, Texas, USA

**Keywords:** anterior cruciate ligament, anterior cruciate ligament reconstruction surgery, autograft, biexponential decay, graft remodeling, knee, knee laxity, ligamentization, magnetic resonance imaging, monoexponential decay, ultrashort echo time T_2*_.

## Abstract

**Background:**

The anterior cruciate ligament reconstruction (ACLR) graft undergoes a remodeling process that affects its structural properties. Ultrashort echo time T_2*_ (UTE-T_2*_) imaging has been instrumental in examining this process. However, more research is needed on the postoperative relationship between UTE-T_2*_ of the graft and its mechanical properties.

**Purpose:**

To longitudinally examine ACL graft changes after ACLR using UTE-T_2*_ decay coefficients and knee laxity and explore their relationship.

**Study Design:**

Case series; Level of evidence, 4.

**Methods:**

A total of 31 patients who underwent ACLR had magnetic resonance imaging of their knees at 1, 6, and 12 months after surgery using a UTE-T_2*_ sequence. Bilateral knee laxity was measured at 6 and 12 months using a GNRB arthrometer (force = 200 N). UTE-T_2*_ coefficients of the graft were calculated using mono- (T_2m*_) and biexponential (short [T_2s*_] and long [T_2l*_]) analyses, and outcomes were normalized to body mass index. Linear mixed models were used to determine longitudinal changes in UTE-T_2*_ and laxity; the Pearson correlation was used to explore the correlations between these outcomes.

**Results:**

T_2m*_ of the graft increased from 1 to 6 months (Δ = 0.092; *P* = .008), followed by a decrease from 6 to 12 months (Δ = −0.079; *P* = .021). Regardless of the limb side, a decrease in laxity was detected between 6 and 12 months after surgery (Δ = −0.033; *P* = .046). Positive correlations between laxity and UTE-T_2*_ were detected at 6 months (T_2s*_: *R* = 0.285; *P* = .025) and 12 months (T_2m*_: *R* = 0.532; *P* < .01; T_2s*_: *R* = 0.669; *P* < .001; T_2l*_: *R* = 0.354; *P* = .034).

**Conclusion:**

Biexponential analysis of UTE-T_2*_ MRI provides a sensitive tool for detecting structural changes in the graft after ACL reconstruction, reflecting the dynamic process of graft remodeling. Among the decay coefficients assessed, T_2s*_ demonstrates a stronger correlation with postoperative laxity, highlighting its potential as a critical biomarker for monitoring graft integrity over time.

Noninvasive assessment of anterior cruciate ligament (ACL) graft maturation and its relationship to its mechanical properties after ACL reconstruction (ACLR) is pivotal in guiding postoperative care. ACLR aims to restore stability and function to the knee joint, typically using autologous grafts harvested from the patellar, quadriceps, or hamstring tendons. When implanted, the graft undergoes the process of ligamentization, where it progressively sheds its initial composition to adopt biomechanical and biochemical traits akin to the native ACL.^2,8,13,19,22,33,51^ Although definitive consensus is lacking on trajectory, timing, and individual variations of its maturation process,^13^ it is generally observed that the ACL graft is most differentiated at 6 months after surgery, begins to stabilize as early as 12 months, and fully matures by 18 to 48+ months.^52^ Identifying objective, noninvasive biomarkers to gauge graft maturation and the relation to its mechanical strength is essential for advancing our understanding of the graft healing process. In the long term, the clinical application of such biomarkers could enhance the monitoring and management of graft health, improve patient outcomes, and reduce the likelihood of graft reinjury.

Recent research has highlighted the ability of ultrashort echo time T_2_* (UTE-T_2_*) magnetic resonance imaging (MRI) to monitor the ligamentization process.^12,49,53^ Unlike conventional MRI sequences, UTE-T_2_* imaging uses several short echo time images to capture subtle nuances in connective tissue composition. This allows UTE-T_2_* to detect the rapid decline of the T_2_ signal in tissues with densely organized collagen matrices,^12^ which is otherwise missed by conventional sequences.^4,5^ In addition, quantitative UTE-T_2_* can provide tissue-dependent decay coefficients that are less sensitive to noise, imaging sequences, and scanner/manufacturer variations, which is a significant problem for signal intensity measurements used in conventional sequences.^4,9,10^ These features make quantitative UTE-T_2_* a promising method for objectively monitoring the graft property changes after surgery and comparing the findings across studies and sites.

In UTE-T_2_* imaging, the signal intensity of each image voxel (ie, a 3-dimensional [3D] pixel) demonstrates exponential decay patterns as echo time increases. This decay is conventionally described by a monoexponential coefficient (T_2m_*). However, recent studies have revealed that the decay better follows a biexponential process composed of 2 distinct phases^11,16,21,28,29^: an initial rapid, brief decline (short T_2_* [T_2s_*]), followed by a slower, prolonged decrease (long T_2_* [T_2l_*]). The rapid decay of T_2s_* is associated with tightly bound water content within tissues, while T_2l_* is related to unbound water content.^11,16,30^ Bound water in connective tissues is believed to be mediated by collagen, proteoglycan, and glycosaminoglycan organization/density within the tissue. As these elements (particularly collagen) dictate the graft's mechanical traits, T_2s_* has been shown to relate strongly to the mechanical properties of connective tissues.^21,37^ On the other hand, T_2m_* represents the weighted mean of T_2s_* and T_2l_*^28^ and cannot differentiate between bound and free water components.^36^ Consequently, more studies are promoting the use of a biexponential decay model^11,17,21,28,29^ to more accurately represent this complex decay process in connective tissues.^21,35^

Aside from quantitative MRI, performing noninvasive tests to directly probe the graft's mechanical attributes remains challenging. The Lachman test is commonly used in clinical settings to assess gross knee laxity, monitor surgical success, guide rehabilitation, predict functional outcomes, and evaluate graft failure risk.^31^ While multiple knee structures contribute to knee laxity, cadaveric studies suggest that a significant portion of loading (up to 90%) is endured by the ACL.^7^ Therefore, laxity can be considered a noninvasive and clinically meaningful assessment to gauge the knee's mechanical stability during anterior loading, primarily modulated by the ACL. Previous studies have objectively quantified knee laxity within the first year after ACLR, noting decreases in laxity between 6 and 12 months,^43^ as well as 9 and 12 months.^39^ These reductions in laxity have been partly attributed to the graft's ligamentization process.

If a reliable correlation can be established between UTE-T_2_* coefficients and the mechanical attributes of connective tissues, this imaging modality could serve as a dependable biomarker for monitoring ACL graft healing while providing more nuanced and mechanistic insights into the remodeling process. Promising correlations between UTE-T_2_* readings and the mechanical traits of connective tissues have been reported in cadaveric^3,5,27,38,55^ and animal studies.^7,20,47,50^ However, its translation to in vivo studies in humans throughout the healing process requires further exploration. Investigating the laxity-UTE-T_2_* relationship, especially using biexponential decay analyses, can help determine whether T_2s_* demonstrates a stronger correlation than T_2l_* and T_2m_* with knee laxity,^37^ because of its suggested stronger relationship with the structural properties in connective tissues.

This study aimed to examine the changes in mono- and biexponential decay coefficients within the first year after ACLR (1, 6, and 12 months) and explore their relationship to knee laxity (6 and 12 months). It was hypothesized that (1) UTE-T_2_* coefficients will be the largest at 6 months compared with other time points, (2) laxity will improve (decrease) from 6 to 12 months, and (3) T_2s_* will present the strongest correlation to laxity, followed by T_2m_* and T_2l_*, respectively, at both time points.

## Methods

### Study Participants

The institutional review board at UTHealth-Houston approved this prospective case series (protocol ID: HSC-MS-21-0224). All participants provided written informed consent prior to participation. The study recruited skeletally mature individuals who underwent unilateral primary ACLR using either quadriceps tendon (QuadT) or bone-patellar tendon-bone (BTB) grafts. The attending surgeon (W.L.) determined graft selection and was not influenced by the study. All participants followed a standardized postsurgical rehabilitation protocol. The exclusion criteria included significant meniscal tears or extensive chondral injuries, infections at the surgical site, previous interventions altering the knee’s natural anatomy, previous orthopaedic surgery related to the knee, ankle, or hip, and any treatment or pathology affecting the knee, including corticosteroid injections within 6 weeks before surgery or high doses of oral or inhaled corticosteroids. A minimum sample size of 27 was determined from an a priori power calculation using a repeated-measures analysis of variance. Input parameters for the calculation were an alpha of .05, a power (1-β) of 0.80, and an expected effect size (η^2^) of 0.06 (ie, medium effect size).

### Study Design

**Figure 1.**
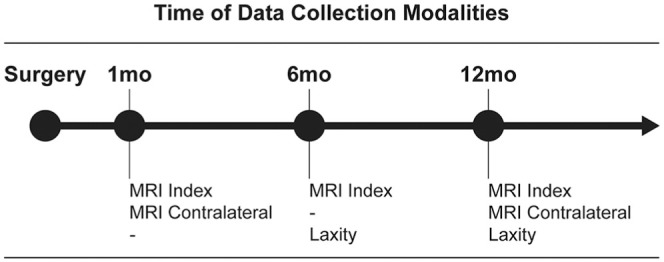
An overview of the study timeline and the data collection modalities used at each time point. MRI, magnetic resonance imaging.

Data were collected at 1, 6, and 12 months after surgery (Figure 1). MRI of the ACLR (index) limb was performed at 1, 6, and 12 months. The contralateral limb was scanned at 1 and 12 months to ensure no changes in the contralateral ACL occurred over time, thus acting as an appropriate internal control. Bilateral laxity measurements were performed at 6 and 12 months.

### Lachman Test

**Figure 2.**
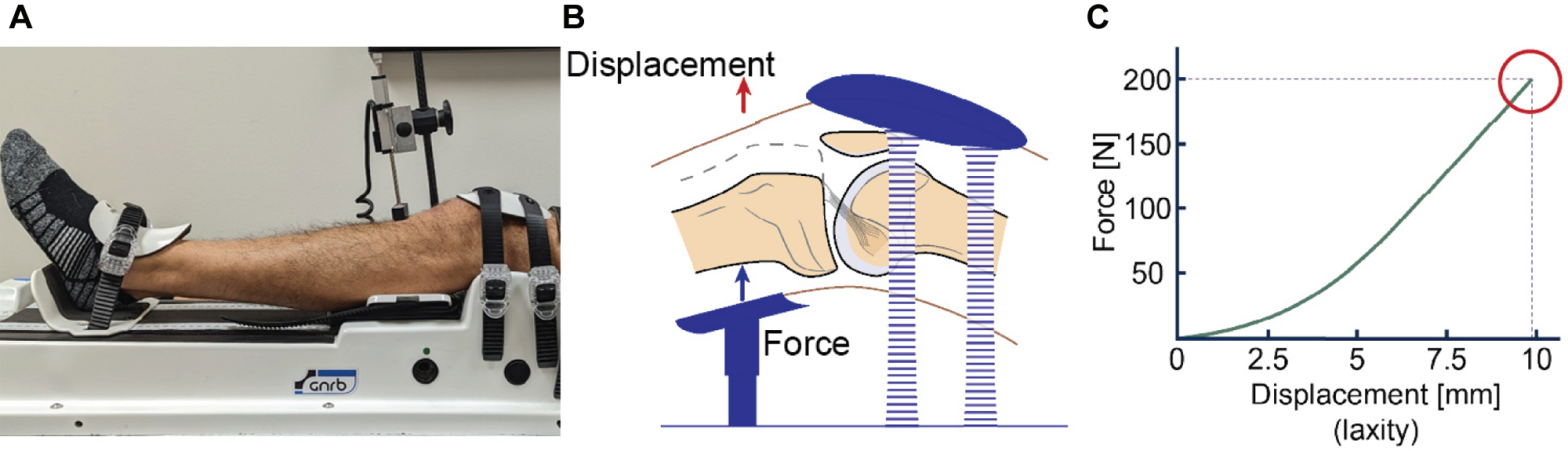
Laxometry measurement using the GNRB arthrometer. (A) The knee was positioned at the standardized position in the GNRB arthrometer, following the manufacturer’s guidelines. (B) An anterior force (0-200 N) was applied to the upper shank, and the corresponding tibial displacements were measured. (C) The acquired force-laxity curve from GNRB laxometry. Displacement values at 200 N (red circle) were used for subsequent analyses of knee laxity.

A mechanically driven Lachman test was performed bilaterally using a GNRB arthrometer (Genourob Inc) (Figure 2) because of its superior reported accuracy^26^ and enhanced objectivity, reliability, and reproducibility^40^ compared with other competitive arthrometry devices, including the typically used KT1000/2000.

The anterior translation of the tibia (relative to the femur) was measured by adhering to the protocols described by the manufacturer.^15^ Briefly, the participant was laid on an examination table with arms placed beside the body. The knee was positioned at approximately 20° of knee flexion, with the patella facing anteriorly^15^ and the foot placed on the footrest (Figure 2A). After aligning the patella with the knee cup, buckles around the tibia and femur were tightened to secure the joint. The patella was securely fastened with a patellar force of 60 ± 5 N, controlled with an embedded pressure feedback control, to standardize the pressure force exerted on the patella during laxity assessments, per previous studies.^1,44-46^ A linear transducer was placed perpendicular to the tibial tuberosity.

The measurement procedure began with the hamstrings relaxed, using an activation monitor inside the actuator.^40^ An anterior force was applied slowly beneath the upper shank until 200 N was reached (Figure 2B). Simultaneously, the linear transducer recorded tibial translation every 5 N with 0.1 mm precision.^40^ A practice trial was performed on each limb to familiarize the patient with the protocol. Afterward, a total of 3 trials were collected per limb. Knee laxity was measured as the difference in tibial position between 0 N (resting) and 200 N (Figure 2C). The mean value of the trials was calculated for each limb and used for further analyses.

### MR Imaging

MRI procedures were conducted with a 3T scanner (Ingenia 3.0T Version 5.3.1; Philips) and a 16-channel knee coil. Patients were positioned supine with their knees flexed at approximately 20°. Foam padding was placed around the knee to prevent movement during and between MRI sequences, thus minimizing motion-related artifacts.

Two co-registered imaging sequences were acquired in this study (Table 1): (1) a high-definition T_2_-weighted m-Dixon sequence in the sagittal plane to improve image contrast and facilitate segmentation of the intra-articular portion of the ACL,^49^ and (2) a 3D radial UTE-T_2_* sequence featuring 9 UTEs (2.3-18.3 ms in 2 ms increments) to calculate the UTE-T_2_* outcomes.

**Table 1:** Parameters Used for the MRI Sequences*^*a*^*

	T_2_-weighted	UTE-T_2_*
Slice thickness, mm	3	1
No. of slices	28	218
Field of view, mm	160 × 160	140 × 140
TE/TR, ms	100/5545	2.3 to 18.3
Voxel size, mm	0.37 × 0.37 ×3	1 × 1 ×1
Pixel bandwidth, Hz	226.4	949.7
Flip angle, deg	90	10
Scan time, min: sec	4: 20	17: 21

a MRI, magnetic resonance imaging; TE, echo time; TR, repetition time; UTE-T_2_*, ultrashort echo time T_2_*.

### Image Processing

UTE-T_2_* maps were created using a least-squares mean method.^23^ To calculate the decay coefficients, monoexponential (Equation 1) and biexponential (Equation 2) decay models were fit to the signal intensity (*S*) of each voxel of the UTE-T_2_* sequence images at different echo times (*TE*):

( Equation 1) $$S_{m}\left(\mathrm{TE}\right)=\;A_{m}e^{-\mathrm{TE}/T_{2m}^{*}}$$

(Equation 2) $$S_{b}\left(\mathrm{TE}\right)=\;A_{s}e^{-\mathrm{TE}/T_{2s}^{*}}+\;A_{l}e^{-\mathrm{TE}/T_{2l}^{*}\;}$$

where *A* is the amplitude of the respective decay coefficient.

Using the T_2_-weighted images, regions of interest (ROIs) representing the intra-articular portion of the graft (ie, from the tibial to femoral insertion points) were manually segmented in Mimics (Materialise). The ROIs were then co-registered to the UTE-T_2_* maps using a semiautomatic registration process inside Mimics.^50^

Individual UTE-T_2_* outcomes were reported as the median T_2_* value of the voxels within each ROI. For the biexponential decay coefficients, voxels were only included if they complied with the condition 4 × T_2s_* < T_2l_*, as recommended previously.^35^ The interobserver reliability of this processing pipeline was previously assessed for T_2m_* by the current study team, demonstrating good to excellent intraclass correlation coefficients.^49^

### Data Normalization

The laxity and UTE-T_2_* decay coefficients of each participant were normalized to their respective body mass index (BMI) (Equations 3 and 4, respectively).

(Equation 3) $$\mathrm{Laxit}y_{\mathrm{norm}}=\frac{\mathrm{Laxity}}{\mathrm{BMI}}$$

(Equation 4) $$T2_{\mathrm{norm}}^{*}=\frac{T2^{*}}{\mathrm{BMI}}$$

The BMI has been shown to influence knee laxity measurements^1,56^ and affect collagen turnover,^32^ with a higher BMI having been found to reduce the laxity of the knee,^1,56^ potentially affecting the ligamentization process.^18^ Therefore, normalizing BMI was a reasonable approach to mitigate its confounding influence on knee laxity^1,56^ and graft imaging attributes,^32^ particularly when performing longitudinal comparisons.

### Statistical Analyses

The normality of the outcome measures was assessed using a Shapiro-Wilks test. A multivariate linear regression, with sex and graft type as the dependent variables, was applied to find differences between cohorts. Two generalized linear models were utilized in this study. The first model tested differences in BMI-normalized UTE-T_2_* coefficients of the index limb at 1, 6, and 12 months (hypothesis 1). The second model tested differences in BMI-normalized laxity at 6 and 12 months (hypothesis 2), with limb side (index vs contralateral) as a within-subject factor. Finally, a Pearson correlation was used to assess the relationship between laxity and UTE-T_2_* decay coefficients of the index limb (hypothesis 3). The correlation coefficient (*R*) was categorized^42^ as follows: very weak (0 < R < 0.1), weak (0.1 ≤*R* < 0.3), moderate (0.3 ≤*R* < 0.50), strong (0.5 ≤*R* < 0.7), very strong (0.7 ≤ R < 0.9), and perfect correlations (0.9 ≤*R*≤ 1). All statistical analyses were conducted in SPSS 29 (IBM), with a significance level set at .05, followed by a least significant difference correction.

## Results

### Study Participants

A total of 31 participants were recruited for this study (men = 14; women = 17; QuadT = 23; BTB = 8; age = 18 ± 3.7 years at the time of surgery; and BMI = 24.06 ± 4.7 kg/m^2^) (Table 2). No relationships were detected between sex/graft type and the outcome measures (laxity and UTE-T2* decay coefficients) (Appendix). Therefore, data from all participants were combined for statistical analyses.

**Table 2:** Characteristics of the Study Participants*^*a*^*

Participant Number	Sex	Graft Type	Age at Surgery, Years	BMI, kg/m^2^
1	F	QuadT	16.7	18.2
2	F	QuadT	17.6	21.1
3	F	QuadT	17.2	26.6
4	F	QuadT	17.2	20.1
5	F	QuadT	24.3	36.3
6	F	QuadT	17.5	21.7
7	F	QuadT	15.2	22.9
8	F	QuadT	16.2	19.9
9	F	QuadT	16.2	26.6
10	F	QuadT	14.8	20.4
11	F	QuadT	18	21.3
12	F	QuadT	18.1	21
13	F	QuadT	17.6	21.2
14	F	QuadT	20	26.3
15	F	QuadT	16.3	22.4
16	F	BTB	17.5	24
17	F	BTB	26	32.7
18	M	QuadT	14.7	29.7
19	M	QuadT	14.7	19.6
20	M	QuadT	14.2	18
21	M	QuadT	23.8	23.7
22	M	QuadT	17.2	22
23	M	QuadT	16.6	22.3
24	M	QuadT	32	25
25	M	QuadT	15.1	17.8
26	M	BTB	17	26.8
27	M	BTB	18.2	26.5
28	M	BTB	16.1	35.9
29	M	BTB	16.7	28
30	M	BTB	16.6	25.1
31	M	BTB	16.3	22.8
Mean Demographic Characteristics				
n	Sex	Graft Type	Age at Surgery, Years	BMI, kg/m^2^
31	17 women	23 QuadT	18 ± 3.7	24.06 ± 4.7
	14 men	8 BTB		

a BMI, body mass index; BTB, bone-patellar tendon-bone; QuadT, quadriceps tendon.

### Longitudinal Changes in UTE-T_2_* and Laxity

**Figure 3.**
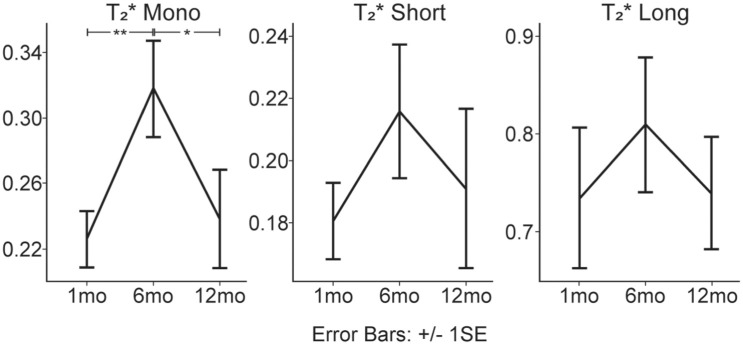
The mean BMI-normalized UTE-T_2_* values at 1, 6, and 12 months after ACLR for the index limb. *indicates *P* < .05; **indicates *P* < .005. ACLR, anterior cruciate ligament reconstruction; UTE-T_2_*, ultrashort echo time T_2_*.

For UTE-T_2_* outcomes in the index limb, a significant increase was detected in T_2m_* from 1 to 6 months (Δ = 0.092; *P* = .008), followed by a decrease from 6 to 12 months (Δ = −0.079; *P* = .021) (Figure 3). Similarly, albeit nonsignificantly, a similar pattern of changes was observed in T_2s_* (1-6 mo: Δ = −0.695; *P* = .100; 6-12 mo: Δ = 0.335; *P* = .376) and T_2l_* (1-6 mo: Δ = −3.416; *P* = .170; 6-12 mo: Δ = 0.676; *P* = .724). For the contralateral limb, no significant differences were detected between 1 and 12 months for all UTE-T_2_* outcomes (T_2m_*: Δ = −0.590; *P* = .347; T_2s_*: Δ = −0.361; *P* = .462; T_2l_*: Δ = −2.740; *P* = .200).

**Figure 4.**
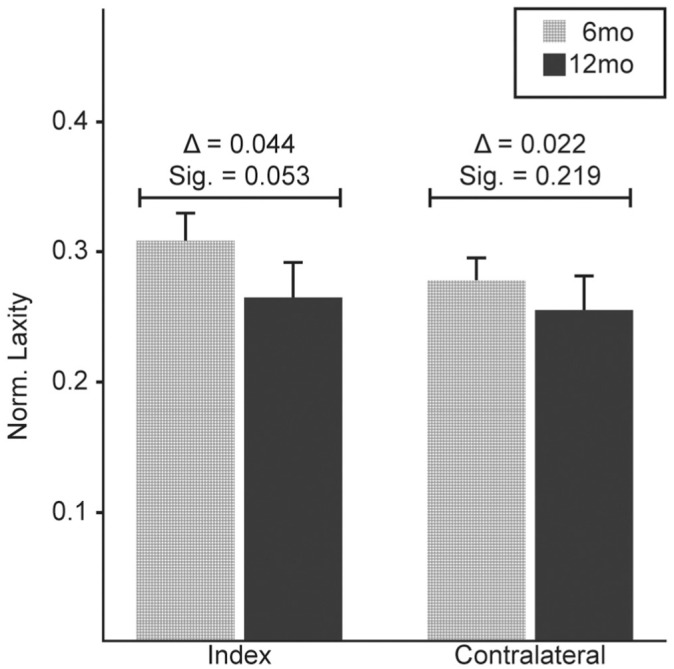
The mean BMI-normalized laxity measures at 6 and 12 months after ACLR for the index and contralateral limb. Error bars represent ± 1 SE. ACLR, anterior cruciate ligament reconstruction; BMI, body mass index; Sig, significant.

No significant longitudinal differences were detected for laxity for both the index (Δ = −0.044; *P* = .053) and contralateral limbs (Δ = −0.022; *P* = 0.219) (Figure 4). However, the index limb demonstrated a trend toward decreased (ie, improved) laxity. Regardless of time, overall laxity was significantly greater in the index limb (Δ = −0.033; *P* = .046).

### Correlations Between UTE-T_2_* and Laxity

**Figure 5.**
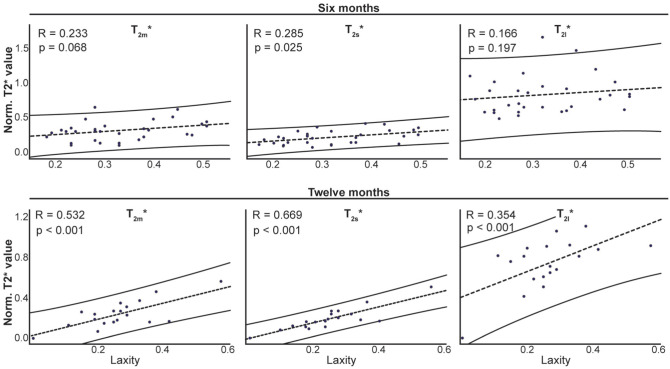
Correlations between BMI-normalized laxity and UTE-T_2_* decay coefficients of the index limb at 6 and 12 months after ACLR. ACLR, anterior cruciate ligament reconstruction; BMI, body mass index; UTE-T_2_*, ultrashort echo time T_2_*.

All correlations between laxity and UTE-T_2_* decay coefficients were positive, indicating that larger laxity values were associated with larger UTE-T_2_* magnitudes (Figure 5). At 6 months, a weak-to-moderate correlation was detected between laxity and T_2s_* (*R* = 0.285; *P* = .025), while T_2m_* and T_2l_* were not correlated with laxity at this time (T_2m_*: *R* = 0.233; *P* = .068; T_2l_*: *R* = 0.166; *P* = .197). At 12 months, strong correlations between T_2s_* and T_2m_* and laxity were detected, with T_2s_* showing the largest correlation (T_2s_*: *R* = 0.669; *P* < .001; T_2m_*: *R* = 0.532; P = .01). A moderate correlation was detected for T_2l_* (*R* = 0.354; *P* = .034).

## Discussion

This study explored the progression and relationship of knee laxity and UTE-T_2_* decay coefficients over the first year after ACLR. Temporal UTE-T_2_* profiles showed a notable peak at 6 months after surgery—a finding consistent with previous research and affirming the first hypothesis.^4,49,52^ Although the laxity findings did not support the second hypothesis, a trend in which the laxity of the index limb decreased from 6 to 12 months warrants discussion. Last, at 6 months, only T_2s_* was significantly correlated with laxity. However, by 12 months, moderate to strong correlations were noted between laxity and all UTE-T_2_* decay coefficients, with T_2s_* showing the strongest correlation compared with other decay coefficients. As such, the third hypothesis was partially supported.

Several key advancements in assessing ACL graft maturation were achieved. While previous research has examined laxity measures and UTE-T_2_* outcomes of connective tissues—such as the in vivo Achilles tendon,^37^ ex vivo porcine ACL,^6^ and in vitro human ACL^11,20,27^—our study is among the first to longitudinally assess the in vivo relationship between ACL graft laxity and biexponential UTE-T_2_* values after surgery. This enabled a detailed investigation of changes in bound and free water content within the graft, providing insights into its structural adaptation over time. The use of a GNRB arthrometer further improved upon the research outcomes when compared with the more commonly used KT-1000/2000, which is impacted by user experience level, differences in patient positioning, and subjective force application by the experimenter,^15^ to name a few. Together, these advancements positioned our study as a novel contribution to understanding ACL graft imaging and mechanical changes after ACLR, with implications for tracking graft health and guiding rehabilitation practices.

### UTE-T_2_* Outcomes Peaked at 6 Months

T_2m_* for the index limb demonstrated significant changes over 12 months, initially increasing from 1 to 6 months by 36.1% before declining by 23.1% from 6 to 12 months. T_2s_* and T_2l_* had a similar trajectory but did not reach significance, demonstrating a 15.4% and 19.7% increase from 1 to 6 months, and a 6.9% and 3.4% decrease from 6 to 12 months, respectively. Furthermore, the lack of significant changes in the contralateral ACL's UTE-T_2_* signal suggests minimal biochemical changes on the uninjured limb during the first year after surgery.

The observed longitudinal changes in decay coefficients align with the proposed “3-stage” framework of the ligamentization process after surgery, which consists of the early, remodeling, and maturation stages.^13,24^ The early stage (0-6 months) involves increased fibroblast activity to prepare the graft for remodeling.^41^ The remodeling stage (3-12 months) is marked by the replacement of mature tendon collagens with an immature ligament-like matrix,^41^ aligning with the present study's observed increase in T_2m_* values from 1 to 6 months. Finally, during the maturation stage (9-48+ months), the collagen network progresses toward maturation into a ligamentous structure^35^ with improved mechanical properties.^6^ The decrease in T_2m_* and laxity from 6 to 12 months in the present study could reflect this transition.

The findings of this study align with previous investigations of T_2m_* in ACL grafts^12,34,49,53^ while also highlighting temporal variations. In another study by this research team on a different cohort, T_2m_* increased by 82% from 1 to 6 months, followed by a 19% decrease from 6 to 12 months, consistent with the current findings.^49^ Similarly, Lansdown et al^34^ observed a significant decrease (~3 ms) in T_2m_* from 6 to 12 months, reinforcing the temporal trends identified here. Conversely, Chu et al^12^ reported a 25% (~4 ms) increase in T_2m_* from 6 weeks to 6 months, stabilization between 6 and 12 months, and a 19% (~3 ms) decrease from 12 to 24 months. These discrepancies may stem from inherent variability in the ligamentization process, variations in patient characteristics (eg, age and activity level), and methodological differences (eg, imaging and surgical techniques).

Nonetheless, the consistent temporal trends reinforce UTE-T_2_* decay coefficients as promising biomarkers for monitoring the graft remodeling, particularly during the critical first year after surgery when decisions about rehabilitation and return to activity are made. Future research should refine these biomarkers and evaluate their broader potential in ACLR care.

### Laxity Improved From 6 to 12 Months

Our findings suggest a trend toward improved laxity in the index limb between 6 and 12 months, although this change fell short of statistical significance. Limited studies investigated the laxity changes within the first year after surgery, incorporating closely spaced follow-up visits. Pouderoux et al^39^ assessed knee laxity at multiple intervals: presurgery, 15 days, and 1, 3, 6, 9, and 12 months after surgery. They observed a rise in relative laxity through the first year, peaking at 9 months, followed by a significant decline and stabilization by 12 months—findings echoed by the earlier work of Semay et al.^43^ These observations align closely with the present study’s findings, demonstrating a reduction in laxity between 6 and 12 months. However, the most significant changes in laxity in these studies unfolded between 9 and 12 months rather than 6 and 12 months.^39^ The absence of measurements at the 9-month mark in the present study likely hampered our ability to observe these intermediate transitions, contributing to the marginal laxity differences between 6 and 12 months. The decision to exclude the 9-month mark was based on a previous MR study,^49^ which longitudinally imaged patients at 1, 3, 6, 9, and 12 months after surgery. We found that T_2_* relaxation peaked at 6 months and then gradually declined by 12 months. This suggested that the mechanical properties of the graft may be weakest and laxity most significant at 6 months, justifying the time points used in this study. Given the apparent dynamic changes in graft properties and knee stability after the 6-month mark, incorporating additional follow-up visits in future studies would provide a more granular evaluation, ensuring that no critical developments remain unexplored.

### Laxity Correlates Strongly to UTE-T_2_*

At 6 months, T_2s_* showed a weak correlation with laxity. At 12 months, all UTE-T_2_* decay coefficients demonstrated a significant correlation, with T_2s_* demonstrating the strongest relationship. Detecting these correlations is pivotal, as it reveals the relationship between the ligamentization process and the mechanical properties of the ligament. This area has been marginally investigated in humans because of the need for invasive biopsy sampling.^14,19,24,25,41,54^

Weak correlations between laxity and T_2s_* at 6 months align with the early/remodeling stage of the ligamentization process, where immature collagen fibers begin to form centers but lack optimal orientation and interconnectivity.^13,19,24,25,41,54^ Between 6 and 12 months, the collagen fibrils continuously evolve to resemble those of a native ACL, but do not fully restore their interconnectivity and strict parallel alignment.^41,50^ It is speculated that this insufficient organization limits the graft's ability to function uniformly and that its mechanical function is contingent upon the weakest collagen connections. Consequently, at 6 months, the graft's mechanical properties may be influenced more by localized deficiencies than its overall biochemical composition, which is represented by UTE-T_2_*, resulting in weaker correlations. By 12 months, as the graft advances into the maturation stage, the collagen network could become more uniformly interconnected and ligamentous.^13,19,24,25,41,54^ This structural homogeneity can be speculated to mitigate the influence of localized imperfections, allowing the graft's gross mechanical properties to align more closely with its average biochemical composition. This progression may explain the stronger correlations observed between UTE-T_2_* and laxity at 12 months. Future studies integrating UTE-T_2_* imaging with magnetic resonance elastography could validate whether UTE-T_2_* outcomes correlate with the elastic properties of the graft on a voxel-by-voxel basis.

T_2s_* exhibited the strongest correlation with knee laxity at both time points, reflecting its sensitivity to bound water—a key determinant of collagen organization and tissue mechanical properties.^14^ In contrast, T_2l_* showed the weakest correlation, consistent with its representation of unbound water, which reflects tissue hydration status and has minimal effect on graft mechanics. T_2m_*, combining both bound and unbound water, displayed intermediate correlations, as expected. These findings position T_2s_* as a promising noninvasive biomarker for monitoring a graft's mechanical function during the ligamentization process.

Previous studies support these findings. Chang et al^11^ demonstrated significant correlations between histological quality and UTE-T_2_* bicomponent outcomes in Achilles tendons, while Wang et al^48^ observed correlations between T_2m_* and anterior knee laxity measured through a KT-2000. Furthermore, Monte et al.^37^ validated the correlation of T_2s_* with the Young modulus in healthy Achilles tendons (*R* = 0.38; *P* = .03), emphasizing the relevance of T_2s_* in characterizing tissue mechanical properties. These findings reinforce the present study’s implications and underscore the potential of T_2s_* in advancing methods of graft assessment.

While the mechanical properties of the graft and UTE-T_2_* outcomes show significant correlations, the utility of UTE-T_2_* imaging extends beyond what simple laxity assessments can offer. Laxity measurements provide functional insights but do not capture the biochemical and structural changes underlying ligamentization. UTE-T_2_* provides a window into the microstructural and biochemical composition of the graft, enabling a detailed evaluation of its remodeling process. This level of granularity can guide not only the monitoring of graft healing but also the optimization of interventions, such as advanced rehabilitation protocols or biological augmentations.

### Strengths and Limitations

Laxometry provides a noninvasive and clinically relevant measure of joint stability, reflecting both graft performance and its interaction with surrounding tissues. Although it does not isolate graft-specific mechanical properties, cadaveric studies showed that the ACL bears up to 90% of anterior loading during sagittal plane movements, highlighting its relevance as a functional outcome.^9^

The small sample size in this study reduces the generalizability of the findings and limits the ability to detect potential differences across factors such as sex and graft type. However, the sample size was determined based on an a priori power calculation that would provide a medium effect size and a power of 0.80. To improve the robustness of future research, larger cohorts should be recruited to enhance statistical power and better account for variability across diverse populations.

An additional limitation was the absence of laxity assessments at the 1-month timepoint, preventing correlation with imaging outcomes during this early stage of recovery. This decision was made deliberately to prioritize patient safety, as applying significant anterior forces to the ACL graft during the early postoperative period could increase the risk of graft injury. Future studies could explore safe methods to assess laxity earlier in the recovery process to capture a more comprehensive description of the ligamentization process.

This study was conducted over 12 months to focus on the ligamentization process during a timeframe considered most critical for clinical decisions. Falconiero et al^19^ analyzed 48 biopsy specimens from human grafts (3-120 months after ACLR). They suggested that remodeling was essentially complete by 12 months, as no significant changes in vascularity or fiber patterns were detected afterward. However, other studies indicate that complete graft remodeling may extend up to 24 months.^25,41,54^ Thus, the outcomes observed at the 12-month mark in this study likely represent an intermediate stage of remodeling rather than full ligamentization. The focus on this timeline was justified by its importance in guiding postoperative rehabilitation and return-to-activity plans. Future follow-up studies on this cohort will examine intermediate and long-term changes in UTE-T_2_* and laxity properties beyond the first year, providing deeper insights into the extended ligamentization process.

## Conclusion

This study observed distinct temporal changes in UTE-T_2_* signals, with a peak at 6 months after surgery, aligning with the ligamentization process. These insights corroborate the existing literature and affirm the relevance of UTE-T_2_* analysis in assessing graft maturation stages. Furthermore, this study highlights UTE-T_2_*, especially T_2s_*, as a valuable biomarker for ACL graft maturation, showing a stronger correlation with laxity, likely because of graft mechanical properties. By examining the laxity-UTE-T_2_* relationship, our findings contribute meaningfully to bridging the gap between mechanical and imaging assessments, supporting the integration of UTE-T_2_* as a relevant tool in patient monitoring and rehabilitation planning after ACLR.

## Supplemental Material

sj-pdf-1-ajs-10.1177_03635465251368393 – Supplemental material for Relationship Between Quantitative MRI UTE T2* of ACL Autografts and BMI-Normalized Knee Laxity within the First Year After ACL Reconstruction Supplemental material, sj-pdf-1-ajs-10.1177_03635465251368393 for Relationship Between Quantitative MRI UTE T2* of ACL Autografts and BMI-Normalized Knee Laxity within the First Year After ACL Reconstruction by Alonso Figueroa, Tomasz Bugajski, Dillon Humpal, Manickam Kumaravel, Walter Lowe and Payam Zandiyeh in The American Journal of Sports Medicine
