# Supplementary material for: Relationship Between Quantitative MRI UTE T2* of ACL Autografts and BMI-Normalized Knee Laxity Within the First Year After ACL Reconstruction
Source: Am J Sports Med. 2025 Sep 19;53(12):2841–50. doi: 10.1177/03635465251368393 (PMC12489169; doi:10.1177/03635465251368393)
Supplement: sj-pdf-1-ajs-10.1177_03635465251368393 – Supplemental material for Relationship Between Quantitative MRI UTE T2* of ACL Autografts and BMI-Normalized Knee Laxity within the First Year After ACL Reconstruction [file sj-pdf-1-ajs-10.1177_03635465251368393.pdf]

Autograft's Quantitative MRI UTE T2* Correlates With BMI-Normalized Knee Laxity In Patients Undergoing Anterior Cruciate Ligament Reconstruction

Appendix

Table A1

Differences in T2* and laxity outcomes dependent on sex and graft type

	1 month laxity			6 month laxity			12 month Laxity		
	β coefficient	*t* Coefficient	*P* Value	β coefficient	*t* Coefficient	*P* Value	β coefficient	*t* Coefficient	*P* Value
Sex	-	-	-	-0.309	-0.625	0.534	-0.202	-0.288	0.775
Graft Type	-	-	-	0.594	0.982	0.330	0.195	0.227	0.822

	T2m*_1mo			T2m*_6mo			T2m*_12mo		
	β coefficient	*t* Coefficient	*P* Value	β coefficient	*t* Coefficient	*P* Value	β coefficient	*t* Coefficient	*P* Value
Sex	-0.244	-0.393	0.696	0.885	0.705	0.487	-0.546	-0.568	0.574
Graft Type	0.656	0.825	0.413	-0.764	-0.497	0.623	0.417	0.354	0.726

	T2s*_1mo			T2s*_6mo			T2s*_12mo		
	β coefficient	*t* Coefficient	*P* Value	β coefficient	*t* Coefficient	*P* Value	β coefficient	*t* Coefficient	*P* Value
Sex	0.252	0.654	0.516	3.000	1.161	0.256	-1.153	-1.907	0.066
Graft Type	-0.112	-0.229	0.820	-0.069	-0.080	0.937	0.849	1.147	0.260

	T2l*_1mo			T2l*_6mo			T2l*_12mo		
	β coefficient	*t* Coefficient	*P* Value	β coefficient	*t* Coefficient	*P* Value	β coefficient	*t* Coefficient	*P* Value
Sex	-0.651	-0.247	0.806	-1.398	-0.552	0.586	-3.661	-1.121	0.271
Graft Type	-0.886	-0.263	0.793	-2.847	-0.919	0.366	-1.797	-0.449	0.656
